# Primary Cutaneous Follicle Centre Lymphoma with Hodgkin and Reed-Sternberg Like Cells: A Case Report and Review of the Literature

**DOI:** 10.1155/2017/9549428

**Published:** 2017-09-26

**Authors:** Fatima A. Aldarweesh, Diana O. Treaba

**Affiliations:** Department of Pathology and Laboratory Medicine, Warren Alpert Medical School of Brown University, Rhode Island Hospital, Providence, RI, USA

## Abstract

An elderly woman with a complex medical history presented with a left forearm mass that slowly developed for several months. The excisional biopsy of this skin mass was remarkable for involvement by a follicle centre cell derived lymphoma with a nodular and diffuse pattern associated with a subset of scattered Hodgkin and Reed-Sternberg like cells. Fluorescence in situ hybridization studies did not detect the presence of IgH-bcl2 fusion transcript, and molecular studies were negative for immunoglobulin heavy chain gene rearrangements and EBV DNA sequences. Hodgkin and Reed-Sternberg like cells are rarely reported in FLs, and the association with primary cutaneous follicle centre lymphoma is extremely rarely seen. To our knowledge, our case is the second case of primary cutaneous follicle centre lymphoma with Hodgkin and Reed-Sternberg like cells.

## 1. Introduction

Follicular lymphoma comprises approximately 20% of all lymphomas in the United States and has a slight female predominance [1, 2]. The most frequent sites of involvement are the lymph nodes, spleen, bone marrow, peripheral blood, and Waldeyer's ring with nonhematopoietic sites such as gastrointestinal tract, soft tissue, skin, ocular adnexa, breast, and testis being also involved, often in the context of systemic disease [2]. Skin involvement has been reported in approx. 4% of the cases [3, 4]. Approximately 90% of follicular lymphoma (FL) cases are characterized by the presence of the *t*(14; 18)(q32; q21). The small subset of *t*(14; 18) negative FL is less well understood and seems to have distinct molecular features including bcl6 rearrangements and trisomy 3 [1, 5]. Primary cutaneous follicle centre lymphoma (PCFCL) is a separate diagnostic entity in the WHO 2008 classification [6] and also in the 2016 WHO criteria revision [7] accounting for approximately 60% of the primary cutaneous B-cell lymphomas [6]. This lymphoma may occur as a solitary plaque or mass, especially involving the head or trunk area, may form a small group of closely located lesions, and rarely may be multifocal. While morphologically and immunophenotypically PCFCL has many similarities with the classic FL, generally they lack bcl2 expression with only a small number of cases being bcl2 positive by immunohistochemistry [8–12] but lacking the IgH-bcl2 fusion. Szablewski and coworkers studied 20 PCFCL cases using BOB1/bcl2 double immunostaining and interphase fluorescence in situ hybridization and concluded that a subset harbors similar genetic alterations with nodal FLs (NFL), including BCL2 breaks and 1p36 deletion [13]. Dissemination to extracutaneous sites occurs in approx. 10% of these patients. Both classical Hodgkin lymphoma and non-Hodgkin lymphomas have been reported in the same person and occasionally FLs have H/RS-like large cells, within the neoplastic nodules or at their periphery [14–21]. The finding of a *t*(14; 18) in a cutaneous FL is a rare event and strongly suggests a secondary NFL.

We present a rare case of IgH-bcl2 negative FL with H/RS cells presenting as a solitary mass on the forearm of an 89-year-old woman. The lack of bcl2-IgH fusion and the absence of lymphadenopathy favor a primary cutaneous follicle centre lymphoma with H/RS cells, a very rare diagnostic entity reported only in one prior case in the English medical literature.

## 2. Report of a Case

**Figure 1:**
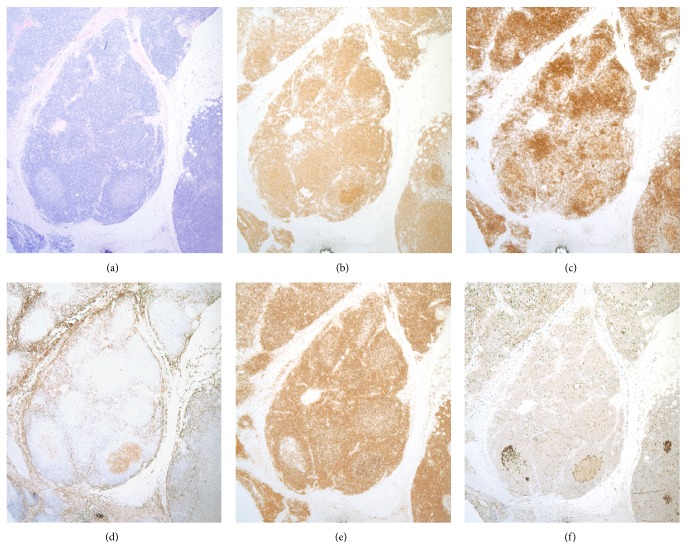
The neoplastic lymphoid follicles ((a), H&E stain, 400x) are of B-cell lineage ((b), CD20 immunostain, 400x), are admixed with a subset of CD3 positive T-cells ((c), CD3 immunostain, 400x), coexpress CD10 ((d), CD10 immunostain, 400x) and bcl2 positivity ((e), bcl2 immunostain, 400x), and have proliferation rates of approximately 20–30% ((f), MIB-1 immunostain, 400x).

**Figure 2:**
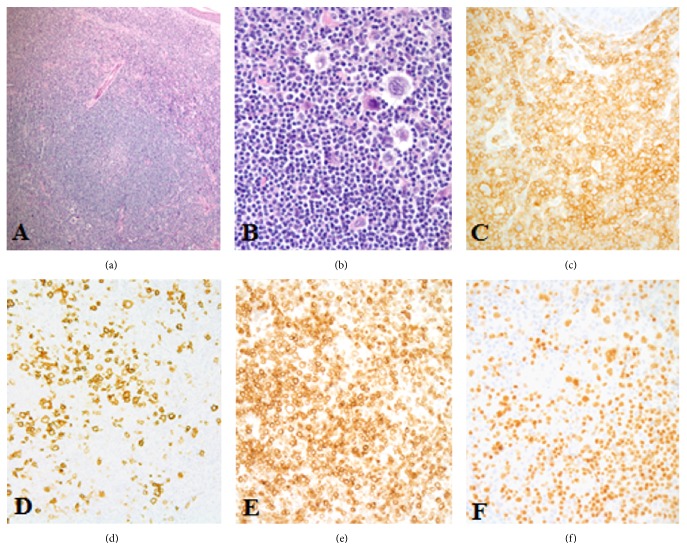
The upper dermis ((a), H&E stain, 400x) has large transformed Hodgkin and Reed-Sternberg like cells ((b), H&E stain, immersion oil 1000x) that coexpress CD45 ((c), CD45 immunostain, 400x), CD30 ((d), CD30 immunostain, 400x), and bcl2 positivity ((e), bcl2 immunostain, 400x) and are also PAX5 positive ((f), PAX5 immunostain, 400x).

An 89-year-old female with a past medical history significant for type 2 diabetes, hypertension, hypothyroidism, coronary artery disease, status after CABG, and hysterectomy presents with a left forearm mass that developed during several months. The patient did not have lymphadenopathy or B-symptoms. The excised 3 × 2.2 × 2 cm mass is remarkable for a dense intradermal lymphoid population with a predominant diffuse pattern of infiltration in the upper dermis and a nodular/follicular pattern in the deeper regions sampled. Some lymphoid follicles had a monotonous appearance of their germinal centres, being composed predominately of centrocytes and only a few centroblasts and lacked tingible body macrophages. In the upper dermis, scattered large transformed lymphoid cells with a Hodgkin and Reed-Sternberg like morphology are identified. By immunohistochemistry, there are neoplastic B-lymphoid follicles coexpressing CD20+, CD10+, bcl6+, and bcl2+ (Figure 1) and exhibiting proliferation rates of approximately 20–30% (MIB-1 antibody), centred by well-developed CD21+ follicular dendritic meshworks. In the upper dermis the H/RS-like cells are noted surrounded by a prominent CD3+, bcl2+ T-cell population. The H/RS-like cells are largely CD45+ and coexpress CD30, CD20, PAX5, bcl2 (Figure 2), CD79a, bcl6, and MUM1 positivity being negative for CD15. An anti-EBV latent membrane protein antibody is negative. FISH studies were negative for IgH-bcl2 fusion. In addition, PCR studies were reported negative for immunoglobulin heavy chain gene rearrangements, T-cell receptor beta, and gamma gene rearrangements and also for EBV DNA sequences.

Given the localized disease, no other treatment was initiated. The patient was lost to follow-up.

## 3. Discussion

The unusual presentation of FLs with H/RS-like cells has been acknowledged in the medical literature mostly in case reports or in review studies and, thus, the true incidence of this association is still unknown. In addition, their presence may be significantly underreported, with the H/RS-like cells being counted as centroblasts in FLs. While their immunophenotype has generally variations from the classic (CD45−, CD30+, PAX5 weak+, and CD15 variably+) Hodgkin lymphoma immunophenotype [22–24], a common germinal centre origin has been suggested for both [22, 25]. In the majority of the reported FL cases their H/RS cells were CD30 positive and coexpressed B-cell markers (CD20, BOB1, PAX5, and OCT.2) and bcl6, were variably CD45 and bcl2 positive, and were less often CD15 or CD10 positive. Similar to classical Hodgkin lymphoma H/RS cells, they can be EBV positive [24]. Furthermore, in some cases of composite lymphomas (CHL and FLs) the H/RS cells of the CHL carried the same IgH-bcl2 translocation as the associated FL, further suggesting a common cell of origin [25–27].

Primary cutaneous follicle centre lymphoma (PCFCL) is most often noted in middle aged to elderly patients, slightly more frequent in men, and has predilection for the head, neck, and the trunk regions, forming single or multiple lesions, papules, plaques, or nodules. Morphologically, PCFCL consists of centrocytes and centroblasts and infiltrates usually the lower half of the dermis and the subcutaneous regions with a follicular, follicular and diffuse, or a diffuse pattern. The immunophenotype is reminiscent of the classical FL with expression of bcl6 and variable CD10 positivity with only weak or completely lacking (in 70% of the cases) bcl2 expression. The absence of bcl2 is also noted at a molecular level, with PCR studies of the PCFCL being virtually always *t*(14; 18) negative. However, the presence of the *t*(14; 18) has been occasionally reported by FISH analysis [28]. The lack of immunoglobulin heavy chain gene rearrangement detection by PCR as seen in our case is generally not unusual for low-grade FLs and often is attributed to an associated large amount of polytypic B-lymphoid cells present in the sample [29].

In our review of the medical literature we identified a single case of PCFCL case with numerous H/RS-like cells positive for CD30, CD15, PAX5, Bcl6, OCT2, BOB1, MUM1, and Ki67 while CD20 was only focally positive. The underlying PCFCL cells expressed CD20, CD79a, bcl6, and CD10 but interpretation of bcl2 was difficult due to an abundant T-cell background. The proliferative index Ki67 in the PCFCL was estimated at 20%. PCR and FISH studies did not detect the presence of the *t*(14; 18) translocation [30]. The comparison between these two isolated cases may suggest that the PCFCLs associated with H/RS cells have low-proliferation rates and appear clinically indolent. Different than H/RS cells associated with chronic lymphocytic leukemia/small lymphocytic lymphoma, these PCFCL's H/RS-like cells were not associated with positivity for EBV. Furthermore they do no embrace the same immunophenotype as classical H/RS cells being CD45 positive and expressing a strong B-cell antigen repertoire. A larger number of cases and a longer patient's follow-up are however needed to better characterize these morphologically unusual PCFCLs with transformed H/RS-like cells.

## 4. Conclusion

We presented a rare case of a PCFCL associated with H/RS-like cells. Skin involvement by PCFCLs with associated H/RS-like cells has been only rarely reported and may constitute a diagnostic challenge requiring a comprehensive antibody panel and various molecular techniques as well as careful clinical investigation to exclude a *t*(14; 18) negative classical FL.
